# Special Issue “Advances in Natural Active Products Derived from Foods: Antioxidant, Antinociceptive and Anti-Inflammatory Activities”

**DOI:** 10.3390/ijms27031395

**Published:** 2026-01-30

**Authors:** Ettore Novellino

**Affiliations:** Department of Medicine and Surgery, Università Cattolica del Sacro Cuore, 00168 Rome, Italy; ettore.novellino@unicatt.it

In recent years, the study of naturally derived bioactive compounds present in foods has attracted increasing interest, owing to their promising potential in preventing and counteracting a wide range of health-related conditions [1,2]. These bioactive compounds, particularly those endowed with antioxidant, antinociceptive, and anti-inflammatory properties, offer promising prospects in both preventive and therapeutic settings [3,4]. This Special Issue is devoted to the investigation of the complex interactions between these compounds and their physiological effects, with particular emphasis on their role in modulating cellular processes and in reducing oxidative stress, pain, and inflammation. Accordingly, the contributions presented in this Special Issue highlight the multifactorial nature of these bioactive agents and underscore their broad potential in clinical applications and public health. Recent evidence suggests that the health-promoting effects of food-derived bioactive compounds are mediated through the modulation of multiple interconnected biological pathways, including redox homeostasis, inflammatory signaling, mitochondrial function, and cellular stress responses [5]. Rather than acting through a single molecular target, these compounds often exert pleiotropic effects that enable them to influence complex pathological processes underlying chronic and degenerative diseases. This multitarget behavior is increasingly recognized as a key advantage in the prevention and management of multifactorial disorders, supporting the growing interest in nutraceutical and functional food-based strategies as complementary approaches to conventional therapies [6]. An overview of the contributions included in this Special Issue is provided in Table 1. A first central theme concerns the neuroprotective and regenerative role of naturally derived compounds, with particular emphasis on mechanisms of neuronal repair and protection against oxidative stress. In this context, it is demonstrated that a nutraceutical based on ursolic acid extracted from agri-food byproducts can promote nerve regeneration and counteract muscle atrophy in models of peripheral nerve injury, highlighting the value of valorizing food byproducts as sources of bioactive molecules with high biological efficacy [7]. In parallel, innovative formulation strategies, such as the prodrug approach and encapsulation in lipid-based systems, emerge as promising tools to enhance the bioavailability and neuroprotective efficacy of natural antioxidant compounds, including ferulic acid and geraniol. A second thematic area focuses on the anti-inflammatory and antioxidant activity of phytocompounds in the context of inflammatory diseases, with particular reference to the skin and peripheral tissues. The effectiveness of plant extracts rich in phenolic compounds and anthocyanins in reducing inflammatory responses and oxidative stress is clearly demonstrated in preclinical models of inflammatory dermatitis, opening up concrete perspectives for the development of natural-based topical formulations aimed at the management of chronic or iatrogenic inflammatory conditions. Another highly relevant theme is represented by the modulation of systemic metabolic axes, particularly the liver–intestine and the gut–cardiovascular system. The reviews included in this Special Issue highlight how bioactive substances present in spices can selectively intervene in mechanisms of oxidative stress, inflammation, and intestinal dysbiosis associated with chronic alcohol consumption, thereby contributing to the restoration of metabolic and immune homeostasis. In a complementary manner, the potential of unconventional functional foods, such as an edible bird’s nest, is discussed in relation to the reduction in major cardiovascular risk factors through the regulation of lipid metabolism, insulin sensitivity, and chronic inflammatory processes. Finally, a cross-cutting theme that strongly emerges is the multifactorial nature of the action of bioactive compounds, which is particularly evident in the context of musculoskeletal and degenerative disorders. Ginsenosides, the main active metabolites of ginseng, exhibit an integrated action across multiple cell types and signaling pathways, modulating inflammatory, oxidative, and tissue remodeling processes in conditions such as osteoporosis, periodontal disease, and osteoarthritis, thereby reinforcing the concept of a systemic and multitarget nutraceutical approach. Overall, the contributions collected in this Special Issue confirm that food-derived bioactive compounds represent a strategic resource for human health, not only because of their antioxidant and anti-inflammatory properties but also due to their ability to act at multiple biological levels, thereby opening up new perspectives for the integration of nutrition, prevention, and therapy. This Special Issue serves as a platform to highlight innovative and ongoing research on the applications of natural food-derived bioactive compounds. The studies presented emphasize the potential of these compounds to play a key role in health prevention, offering alternatives or complements to conventional therapies. We hope that the findings reported in this Special Issue will stimulate further investigations into the therapeutic use of natural compounds and foster collaborations across diverse research fields to address the complex challenges of human health.

## Figures and Tables

**Table 1:** Overview of the articles published in the Special Issue “Advances in Natural Active Products Derived from Foods: Antioxidant, Antinociceptive and Anti-inflammatory Activities”.

Authors	Article Type	Main Topic
Iannuzzo et al.	Original Article	Ursolic acid and nerve regeneration
Lerin et al.	Original Article	Lipid-based delivery of ferulic acid
Lee et al.	Original Article	Phenolic-rich extracts in dermatitis
Huang et al.	Review Article	Spice bioactives and liver–intestine axis
Rusanuar et al.	Review Article	Edible bird’s nest and cardiovascular health
Ko et al.	Review Article	Ginsenosides and musculoskeletal health
